# Supplementary material for: A New Fungal Triterpene from the Fungus Aspergillus flavus Stimulates Glucose Uptake without Fat Accumulation
Source: Mar Drugs. 2022 Mar 10;20(3):203. doi: 10.3390/md20030203 (PMC8953101; doi:10.3390/md20030203)
Supplement: Supplementary file 1 [file marinedrugs-20-00203-s001.zip › marinedrugs-1609319-supplementary.pdf]

A new fungal triterpene from the fungus *Aspergillus flavus* stimulates glucose uptake without fat accumulation

Dan-dan Li[1], Ying Wang[1], Eun La Kim[1], Jongki Hong[2] and Jee H. Jung[1,*]

[1] College of Pharmacy, Pusan National University, 46241 Busan, Republic of Korea; 18840659614@163.com (D.-D.L.); 15521334275@163.com (Y.W.); eunlakim@gmail.com (E.L.K.); jhjung@pusan.ac.kr (J.H.J.)

[2] College of Pharmacy, Kyung Hee University, 02447 Seoul, Republic of Korea; jhong@khu.ac.kr (J.H.)

*Correspondence: jhjung@pusan.ac.kr (J.H.J)

Supplementary Experimental Section

6,8-Dihydroxy-3-((1*E*,3*E*)-penta-1,3-dien-1-yl) isochroman-1-one (**2**): 2.4 mg. ^{1}H NMR (500 MHz, CD_3OD): δ 6.40, (dd, J = 15.2, 10.5 Hz, 1H), δ 6.23 (s, 1H), δ 6.20 (d, J = 1.9 Hz, 1H), δ 6.15-6.08 (m, 1H), δ 5.84 (dq, J = 13.6, 6.7 Hz, 1H), δ 5.70 (dd, J = 15.3, 6.9 Hz, 1H), δ 5.06 (dt, J = 12.4, 6.2 Hz, 1H), δ 3.02-2.91 (m, 2H), δ 1.78 (d, J = 6.4 Hz, 3H). ^{13}C NMR (100 MHz, CD_3OD): δ 171.40, 166.50, 165.51, 143.10, 135.36, 133.04, 131.62, 127.59, 108.09, 102.27, 101.56, 80.73, 34.36, 18.25.

1-(4-Hydroxy-2-methoxyphenyl)-1-propanone (**3**): 1.74 mg. ^{1}H NMR (500 MHz, CD_3OD): δ 7.60-7.56 (m, 1H), δ 7.55 (s, 1H), δ 6.86 (d, J = 8.2 Hz, 1H), δ 3.92 (s, 3H), δ 3.00 (q, J = 7.3 Hz, 2H), δ 1.18 (t, J = 7.3 Hz, 3H) ppm. ^{13}C NMR (100 MHz, CD_3OD): δ 201.01, 152.23, 147.97, 129.04, 123.17, 114.49, 110.44, 55.10, 30.09, 8.44.

Phomapyrone C (**4**): 1.56 mg. $[\alpha]_{D}^{26}$ = +8.4 (1 mg/mL, MeOH). ^{1}H NMR (500 MHz, CD_3OD): δ 5.95 (s, 1H), δ 2.51-2.43 (m, 1H), δ 1.85 (s, 3H), δ 1.69 (dt, J = 14.6, 7.5 Hz, 1H), δ 1.56 (dq, J = 13.7, 6.9 Hz, 1H), δ 1.21 (d, J = 6.9 Hz, 3H), δ 0.90 (t, J = 7.4 Hz,

3H) ppm. ^{13}C NMR (100 MHz, CD_3OD): δ 171, 170.0, 167.0, 101.65, 98.0, 40.88, 28.54, 18.27, 11.85, 8.29.

Daidzein (**5**): 2.87 mg. ^{1}H NMR (500 MHz, CD_3OD): δ 8.10 (s, 1H), δ 8.02 (d, J = 8.8 Hz, 1H), δ 7.39-7.35 (m, 2H), δ 6.90 (dd, J = 8.9, 2.2 Hz, 1H), δ 6.86-6.84 (m, 2H), δ 6.78 (d, J = 2.2 Hz, 1H) ppm. ^{13}C NMR (100 MHz, CD_3OD): δ 174, 164, 157, 152, 129, 127, 124, 123, 116, 115, 114, 101.

Genistein (**6**): 5.65 mg. ^{1}H NMR (500 MHz, CD_3OD): δ 8.04 (s, 1H), δ 7.37 (d, J = 8.1 Hz, 2H), δ 6.84 (d, J = 8.1 Hz, 2H), δ 6.33 (d, J = 2.2 Hz, 1H), 6.22 (d, J = 2.0 Hz, 1H) ppm. ^{13}C NMR (100 MHz, CD_3OD): δ 182.26, 165.95, 163.87, 159.73, 158.83, 154.80, 131.38, 124.75, 123.30, 116.26, 106.29, 100.11, 94.76.

(*S*)-(+)-1-(4-Hydroxy-2,6-dimethoxy-3,5-dimethylphenyl)-2-methylbutan-1-one (**7**): 1.04 mg. $[\alpha]_D^{26}$ = +0.94 (1 mg/mL, MeOH). ^{1}H NMR (500 MHz, CD_3OD): δ 3.65 (s, 6H), δ 2.93 (td, J = 7.1, 6.0 Hz, 1H), δ 2.11 (s, 6H), δ 1.76 (dqd, J = 13.5, 7.5, 5.9 Hz, 1H), δ 1.42-1.32 (m, 1H), δ 1.10 (d, J = 7.0 Hz, 3H), δ 0.93 (t, J = 7.4 Hz, 3H) ppm. ^{13}C NMR (100 MHz, CD_3OD): δ 211, 157, 155, 123, 115, 62.8, 50.3, 26.45, 15.72, 11.94, 9.29.

1-Acetyl-β-carboline (**8**): 0.66 mg. ^{1}H NMR (500 MHz, CD_3OD): δ 8.48 (d, J = 4.9 Hz, 1H), δ 8.33 (d, J = 4.9 Hz, 1H), δ 8.24 (d, J = 7.9 Hz, 1H), δ 7.72 (d, J = 8.3 Hz, 1H), δ 7.61 (t, J = 7.7 Hz, 1H), δ 7.33 (t, J = 7.5 Hz, 1H), δ 2.84 (s, 3H) ppm. ^{13}C NMR (100 MHz, CD_3OD): δ 203.19, 143.38, 138.63, 137.21, 136.12, 133.16, 130.30, 122.65, 121.61, 120.19, 113.48, 26.06.

MK800-62F1 (**9**): 2.7 mg. ^{1}H NMR (500 MHz, CD_3OD): δ 5.22 (s, 1H), δ 4.26 (d, J = 7.5 Hz, 1H), δ 4.12 (d, J = 11.1 Hz, 1H), δ 3.89-3.83 (m, 1H), δ 3.76 (d, J = 3.6 Hz, 1H), δ 3.63 (dd, J = 9.3, 7.5 Hz, 1H), δ 3.54 (d, J = 3.2 Hz, 1H), δ 3.53-3.48 (m, 2H), δ 3.41 (d, J = 11.1 Hz, 1H), δ 3.38-3.32 (m, 3H), δ 2.19 (dd, J = 13.7, 4.2 Hz, 1H), δ 1.98-1.86 (m, 5H), δ 1.83-1.74 (m, 1H), δ 1.72-1.53 (m, 5H), δ 1.48-1.33 (m, 2H), δ 1.22 (s, 3H), δ 1.19 (s, 3H), δ 1.18-1.14 (m, 1H), δ 1.08-0.97 (m overlap, 3H), δ 1.04 (s,

3H), δ 1.02 (s, 3H), δ 1.01 (s, 3H), δ 0.97 (s, 3H), δ 0.95 (s, 3H), δ 0.89 (d, J = 11.9 Hz, 2H). ^{13}C NMR (100 MHz, CD_3OD): δ 145.09, 123.53, 108.79, 93.00, 81.24, 76.89, 75.32, 73.88, 70.18, 67.68, 65.29, 57.18, 49.09, 48.01, 45.29, 43.55, 42.73, 41.22, 40.00, 39.68, 37.82, 37.15, 34.01, 31.31, 28.74, 28.61, 27.34, 27.01, 24.86, 23.22, 23.20, 21.24, 19.8, 17.34, 16.5.

Sigmoside C (**10**): 1.5 mg. ^{1}H NMR (500 MHz, CD3OD): δ 5.24 (t, J = 3.6 Hz, 1H), δ 4.55 (s, 1H), δ 4.13 (d, J = 11.0 Hz, 1H), δ 3.87 (dd, J = 11.8, 2.4 Hz, 1H), δ 3.80 (d, J = 3.2, 1H), δ 3.74 (dd, J = 11.7, 5.5 Hz, 1H), δ 3.58-3.53 (m, 2H), δ 3.46 (dd, J = 9.4, 3.3 Hz, 1H), δ 3.40 (d, J = 11.1 Hz, 1H), δ 3.38-3.31 (m overlap, 1H), δ 3.18 (ddd, J = 9.8, 5.6, 2.5 Hz, 1H), δ 2.22 (d, J = 13.3 Hz, 1H), δ 1.94-1.90 (m, 2H), δ 1.87-1.76 (m, 4H), δ 1.71-1.65 (m, 3H), δ 1.63-1.53 (m, 3H), δ 1.47-1.37 (m, 3H), δ 1.22 (s, 3H), δ 1.17 (s, 3H), δ 1.05 (s, 3H), δ 1.01 (s, 3H), δ 1.06-0.97(m overlap, 3H), δ 0.97 (s, 3H), δ 0.96 (s, 3H), δ 0.92 (s, 3H), δ 0.90-0.87 (m, 1H). ^{13}C NMR (100 MHz, CD_3OD): δ 145.72, 123.26, 98.57, 82.33, 81.26, 78.22, 75.66, 73.10, 68.83, 65.31, 62.93, 57.24, 49.2, 47.45, 46.16, 43.57, 43.05, 41.06, 39.73, 38.17, 37.85, 37.22, 34.28, 33.66, 31.39, 28.8, 28.5, 28.37, 27.10, 26.35, 24.84, 23.21, 21.50, 19.81, 17.47, 16.55.

Prosapogenol (**11**): 0.95 mg. ^{1}H NMR (500 MHz, CD_3OD): δ 5.22 (q, J = 3.5 Hz, 1H), δ 4.49-4.45 (m, 1H), δ 4.32 (d, J = 7.8 Hz, 1H), δ 4.13 (d, J = 11.1 Hz, 1H), δ 3.96 (d, J = 3.2 Hz, 1H), δ 3.90-3.83 (m, 2H), δ 3.80-3.74 (m, 1H), δ 3.61 (d, J = 3.1 Hz, 1H), δ 3.54-3.49 (m, 3H), δ 3.41 (d, J = 11.1 Hz, 1H), δ 3.38-3.28 (m overlap, 3H), δ 3.22 (t, J = 10.8 Hz, 1H), δ 2.18 (d, J = 15.2 Hz, 1H), δ 1.98-1.84 (m, 5H), δ 1.79 (q, J = 11.9 Hz, 1H), δ 1.68 (d, J = 13.0 Hz, 3H), δ 1.65-1.52 (m, 3H), δ 1.45 (t, J = 12.6 Hz, 1H), δ 1.40-1.29 (m, 3H), δ 1.22 (d, J = 1.7 Hz, 3H), δ 1.19 (s, 3H), δ 1.18-1.14 (m, 2H), δ 1.04 (s, 3H), δ 1.02 (s, 3H), δ 1.01 (s, 3H), δ 0.97 (d, J = 1.9 Hz, 3H), δ 0.95 (s, 3H), 0.94 (s, 1H), 0.89 (d, J = 11.5 Hz, 1H). ^{13}C NMR (100 MHz, CD_3OD): δ 145.05, 123.55, 108.58, 106.51, 93.79, 85.10, 81.21, 77.72, 77.52, 76.95, 75.22, 72.88, 71.06, 69.88, 67.22, 66.97, 65.29, 57.16, 49.0, 48.0, 45.26, 43.54, 42.71, 41.22, 39.98, 39.67, 37.81, 37.19, 33.99, 31.33, 28.61, 28.34, 27.34, 27.03, 24.85, 23.27, 23.22, 21.16, 19.8, 17.34, 16.49.

Supplementary Results

Primer Information

Sequencing Primer Name Primer Sequences	PCR Primer Name Primer Sequences
ITS1 5' (TCC GTA GGT GAA CCT GCG G) 3'	ITS1 5' (TCC GTA GGT GAA CCT GCG G) 3'
ITS4 5' (TCC TCC GCT TAT TGA TAT GC) 3'	ITS4 5' (TCC TCC GCT TAT TGA TAT GC) 3'

Subject						Score		Identities	
Accession	Description	Length	Start	End	Coverage	Bit	E-Value	Match/Total	Pct.(%)
KY234273.1	Aspergillus flavus	819	4	815	99	1188	0.0	767/823	93

Kingdom	Family	Genus	Species
Eukaryota	-	Aspergillus	Aspergillus flavus

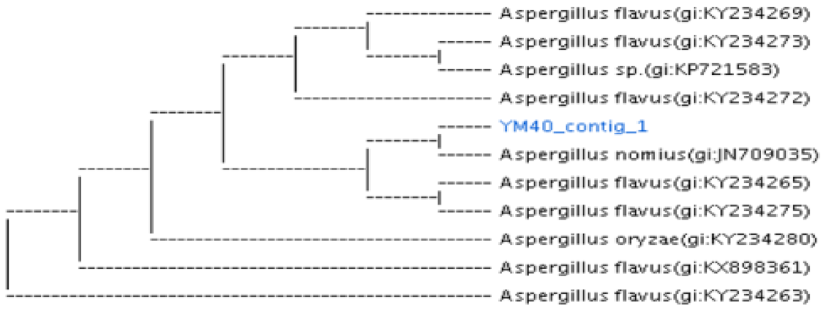

Figure S1: The fungus *Aspergillus flavus* was identified by ITS gene sequences (GenBank accession no. KY234273.1).

Elemental composition	Theoretical m/z	Observed m/z	Error (ppm)
$C_{31}H_{49}O_7$	533.3478	533.3484	+1.13

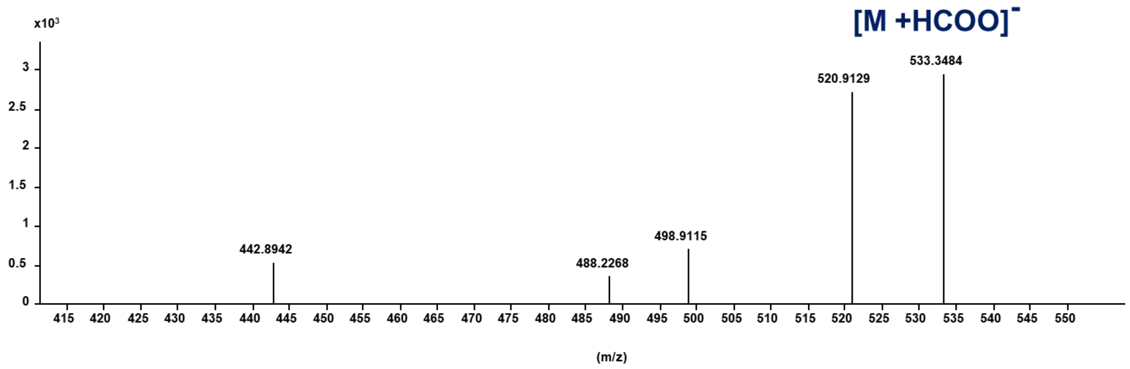

Figure S2: The HRESI mass spectrum of compound **1** measured in negative ion mode.

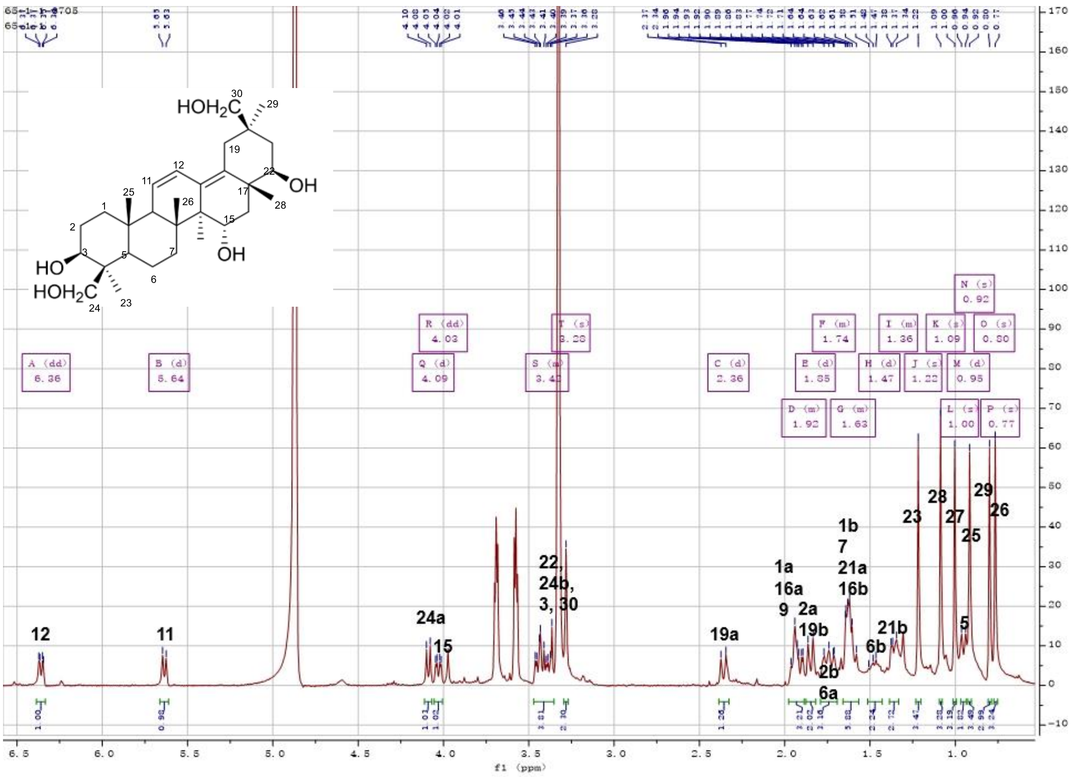

Figure S3: The proton NMR spectrum of compound **1** (500 MHz, CD_3OD). The impurity peaks **(**δ 3.6 and δ 3.7 in ^{1}H NMR spectrum, and δ 62 and δ 73 in ^{13}C NMR spectrum**)** would come from the NMR solvent and appeared in every spectrum. We cannot identify the impurity. The impurity appeared in every spectrum but the bioactivity of tested compounds were quite different to each other. Therefore, we believe that the impurity comes only from NMR solvent and it would not affect the bioassay results

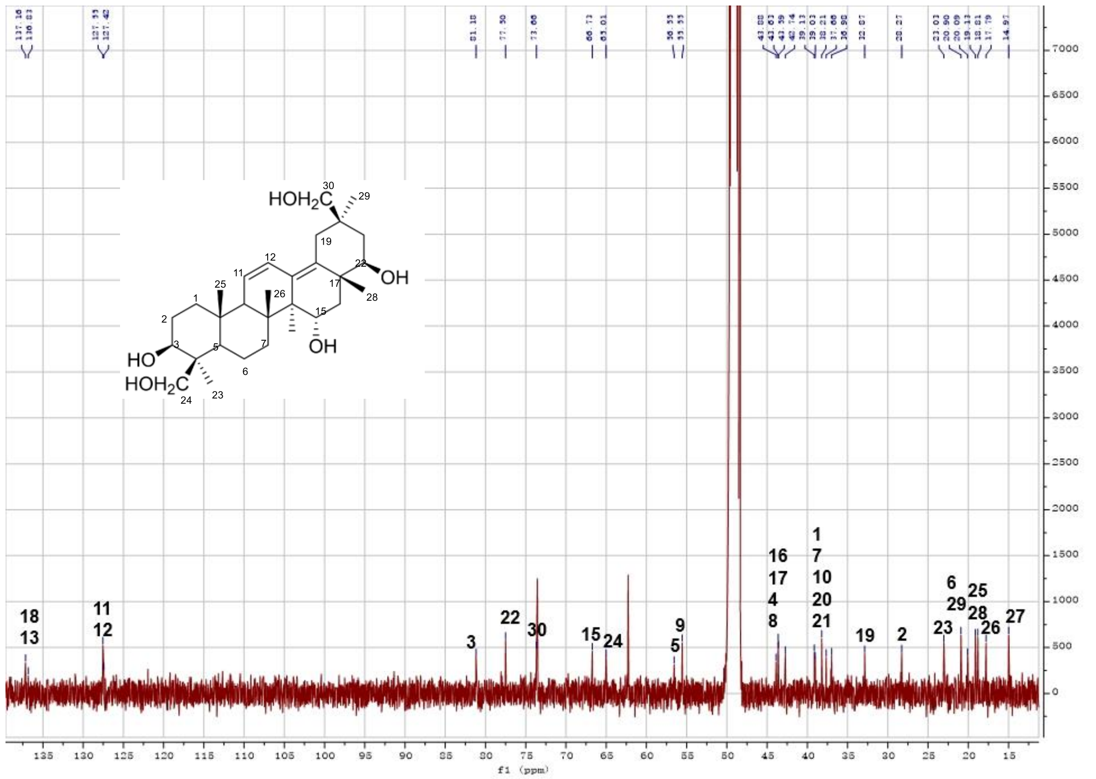

Figure S4: The ^{13}C NMR spectrum of compound **1** (100 MHz, CD_3OD).

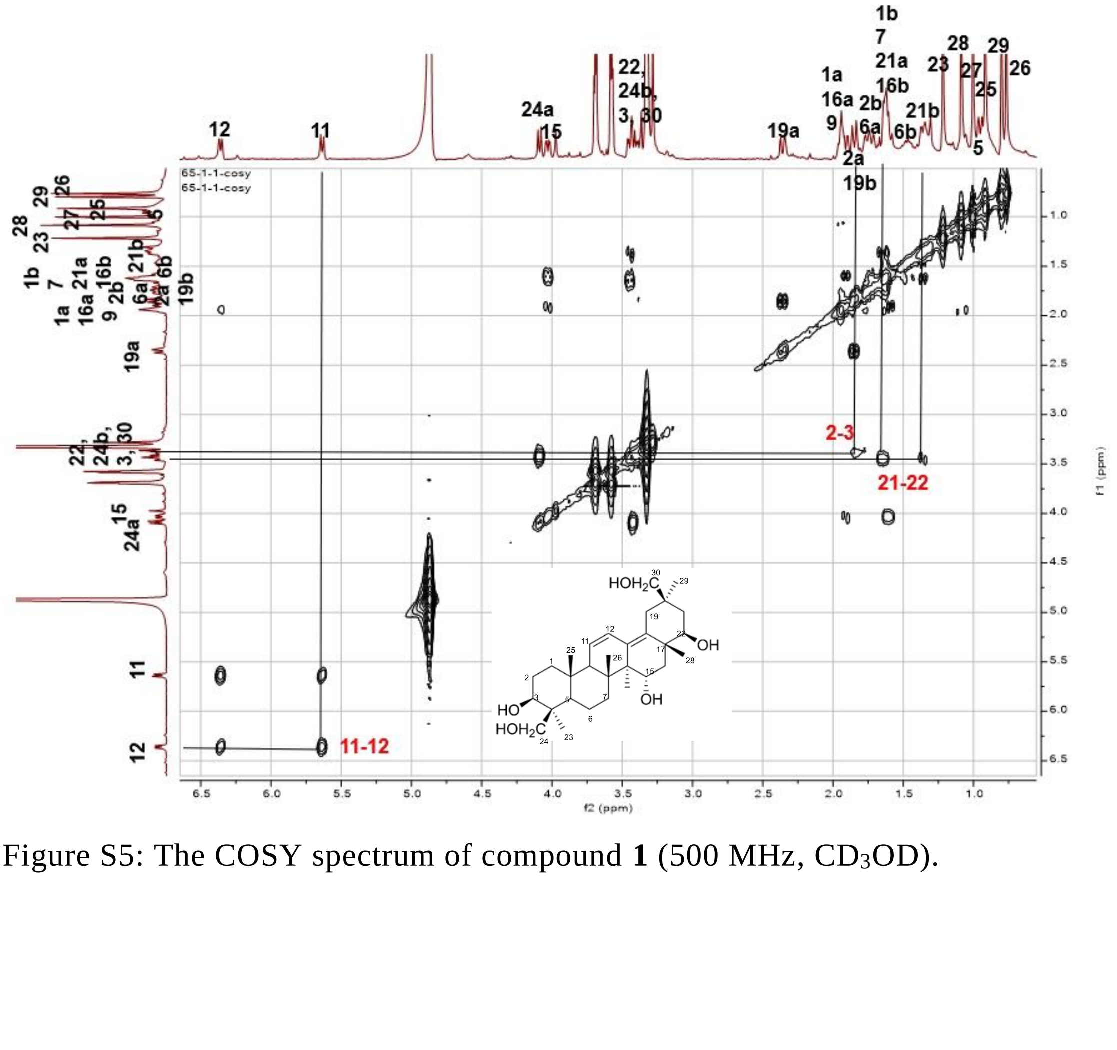

Figure S5: The COSY spectrum of compound **1** (500 MHz, CD_3OD).

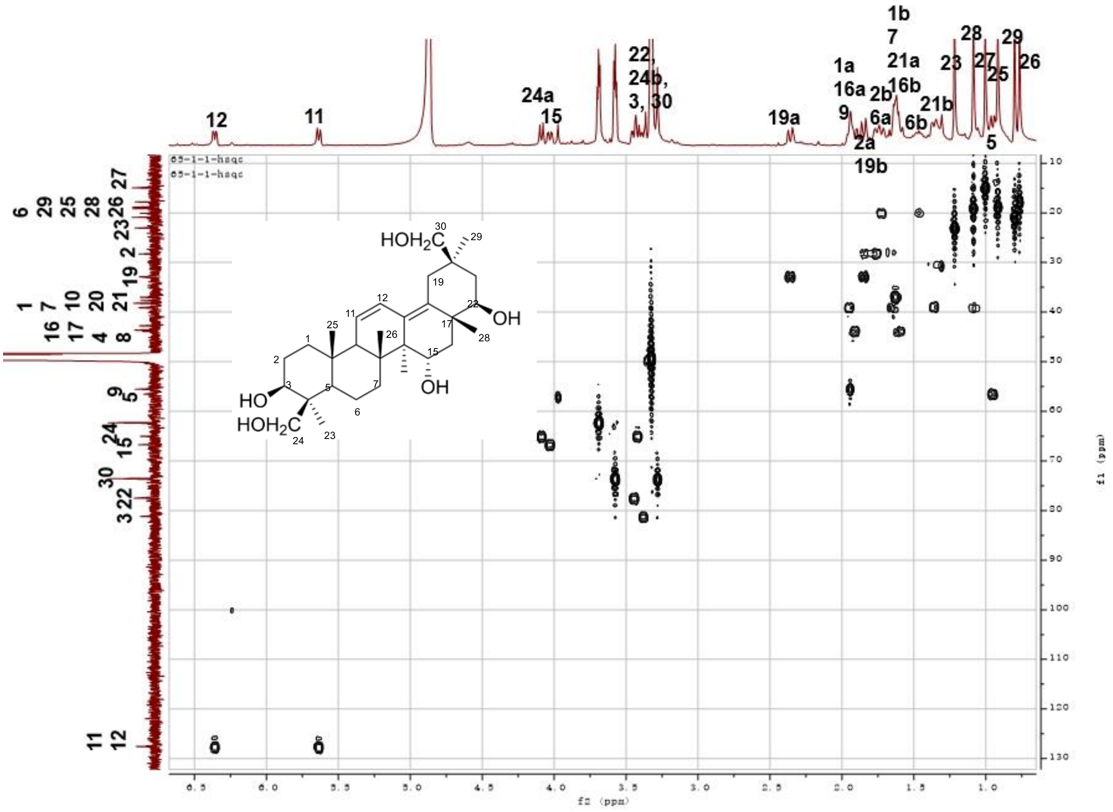

Figure S6: The HSQC spectrum of compound **1** (500 MHz, CD_3OD).

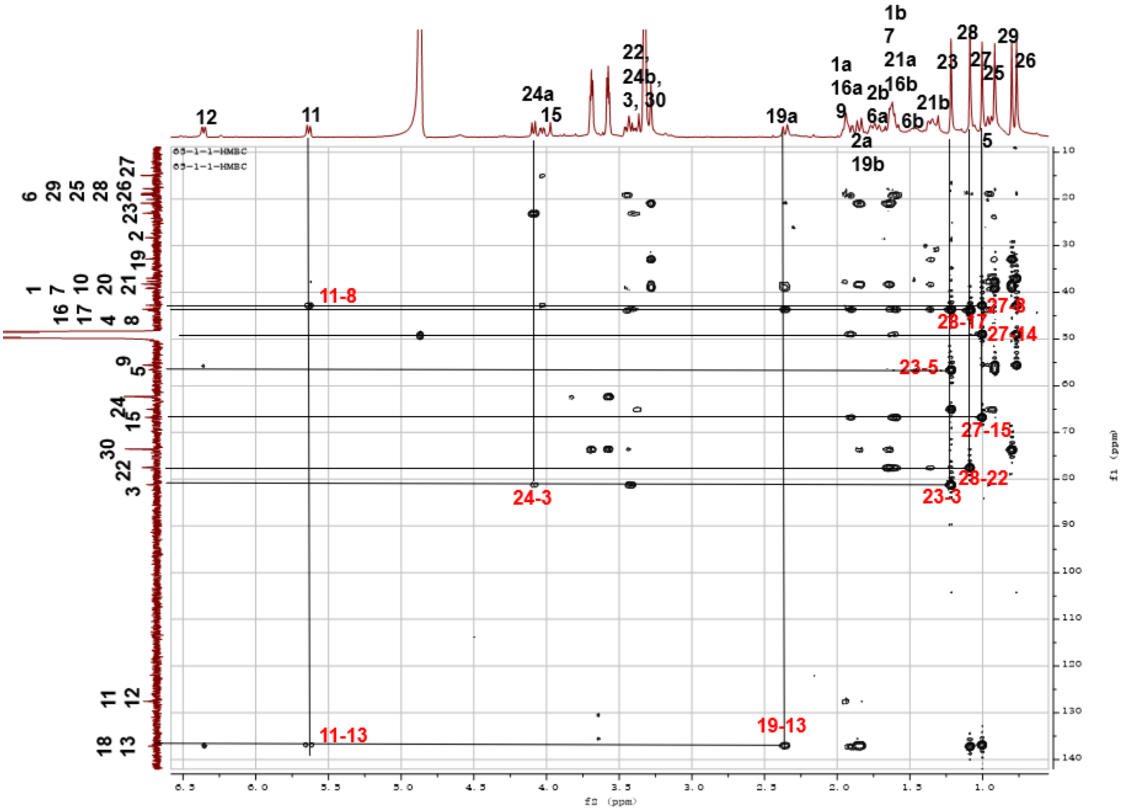

Figure S7: The HMBC spectrum of compound **1** (500 MHz, CD_3OD).

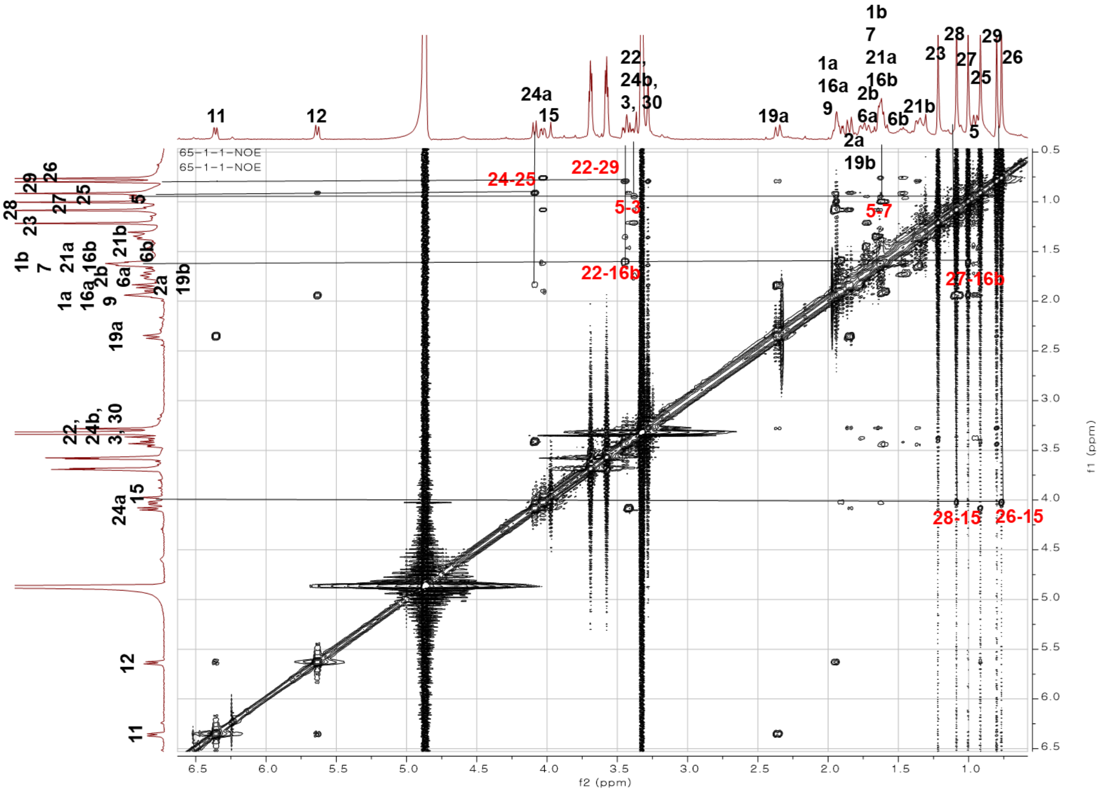

Figure S8: The NOESY spectrum of compound **1** (500 MHz, CD_3OD).

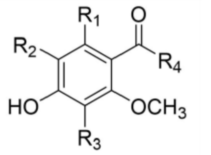

3 R_1 = H, R_2 = H, R_3 = H, R_4 = ethyl
7 R_1 = OCH_3, R_2 = CH_3, R_3 = CH_3, R_4 = *sec*-butyl

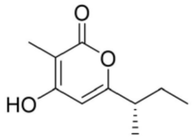

4

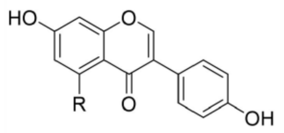

5 R = H
6 R = OH

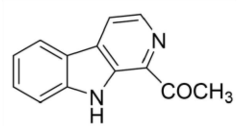

8

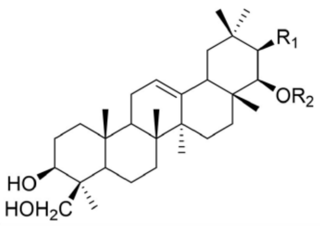

9 R_1 = OH, R_2 = *O*-α-L-arabinopyranosyl
10 R_1 = H, R_2 = *O*-β-D-glucopyranosyl
11 R_1 = OH, R_2 = *O*-[β-D-glucopyranosyl(1-3)-α-L-arabinopyranosyl]

Figure S9. Compounds isolated from the jellyfish-derived fungus *Aspergillus flavus* by activity-guided fractionation.

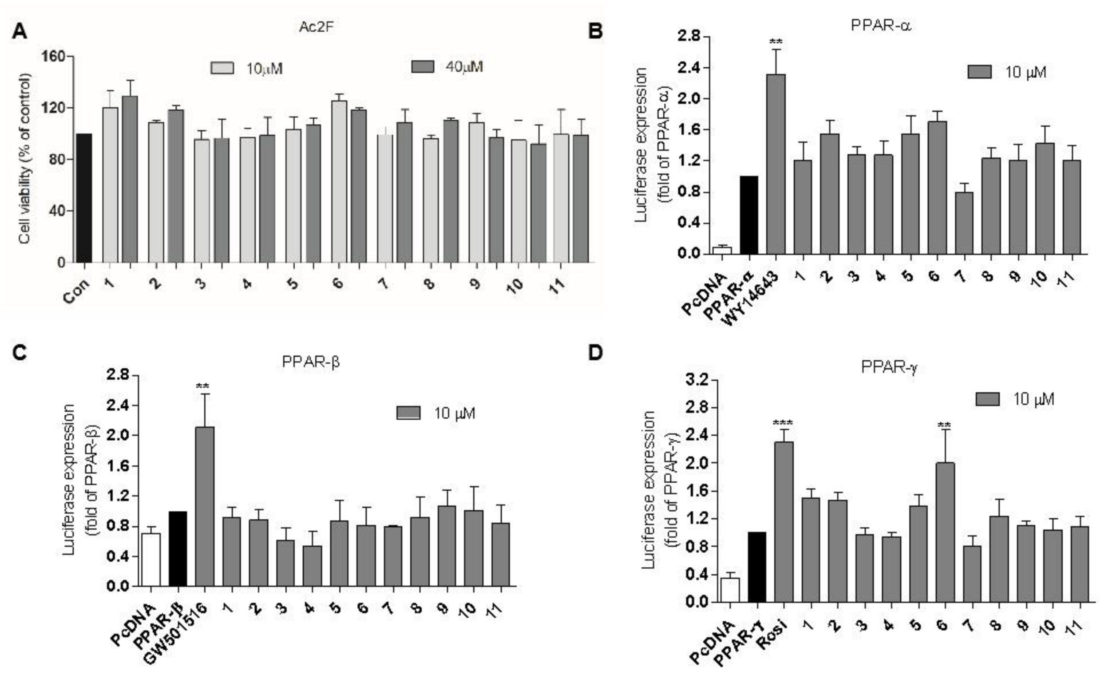

Figure S10: Transactivation effects of PPAR-α, -β/δ, and -ϒ in Ac2F cells when treated with the compounds. (A) Viability of Ac2F cells when treated with the compounds for 12 h. Control group was added only the media and shown as a black bar. (B) The transactivation effect of PPAR-α when treated with the compounds (C) The transactivation effect of PPAR-β/δ when treated with the compounds (D) The transactivation effect of PPAR-ϒ when treated with the compounds. WY-14643, GW501516, and rosiglitazone were used as positive controls for PPAR-α, -β/δ, and -ϒ, respectively. The cells transfected with PcDNA were employed as blank group and shown as empty bars. The cells transfected with PPAR-α, -β/δ, -ϒ and PPRE were employed as control and were shown as black bars. $^{**}P < 0.01$, $^{***}P < 0.001$ vs. PPAR-α, -β/δ or -ϒ.
